# The interpersonal benefits of goal adjustment capacities: the sample case of coping with poor sleep in couples

**DOI:** 10.3389/fpsyg.2024.1287470

**Published:** 2024-03-19

**Authors:** Meaghan A. Barlow, Carsten Wrosch, Christiane A. Hoppmann

**Affiliations:** ^1^Department of Psychology, Wilfrid Laurier University, Waterloo, ON, Canada; ^2^Department of Psychology, Concordia University, Montreal, QC, Canada; ^3^Department of Psychology, University of British Columbia, Vancouver, BC, Canada

**Keywords:** goal adjustment, sleep efficiency, coping, relationship satisfaction, romantic couples

## Abstract

**Introduction:**

This study examined the role of goal adjustment capacities and coping in the association between spousal sleep efficiency and relationship satisfaction in romantic couples.

**Method:**

A community lifespan sample of 113 heterosexual couples (age range = 21–82 years) was recruited using newspaper advertisements in the Greater Montreal Area from June 2011 to December 2012. Participants completed study measures (i.e., Goal Adjustment Scale, Brief Pittsburgh Sleep Quality Index, Relationship Assessment Scale, and the Brief Cope) at two time points, ~1 year apart.

**Results:**

The results of actor-partner interdependence models with moderation (MIXED procedure in SPSS) reveal that goal disengagement buffered people from worsening relationship satisfaction associated with poor spousal sleep [95% CI *B* (−1.17, −0.12)], in part via increases in actor active coping [95% CI *B* (−0.32, −0.02)] and decreases in partner self-blame [95% CI *B* (−0.28, −0.01)]. Goal reengagement was related to diminished relationship satisfaction in response to poor own sleep [95% CI *B* (0.59, 1.79)], in part through increases in actor behavioral disengagement [95% CI *B* (0.05, 0.41)].

**Discussion:**

These findings point to a need for future studies to examine goal adjustment capacities and relationship-specific coping strategies as potential targets of intervention to maintain peoples' relationship satisfaction in the face of sleep problems.

## The interpersonal benefits of goal adjustment capacities: the sample case of coping with poor sleep in couples

The association between goal adjustment capacities and subjective wellbeing is well-established (Barlow et al., 2020). However, stressors that require goal adjustment do not take place in a social vacuum and often involve close others. Yet, research examining the protective effect of goal adjustment capacities in a dyadic context is limited. One prominent stressor that can affect both members of a couple is poor sleep. An emerging body of dyadic research exploring the interpersonal nature of sleep problems suggests that both individual and spousal sleep problems can compromise relationship functioning (Strawbridge et al., 2004; Troxel et al., 2007; Hasler and Troxel, 2010; Troxel, 2010; Gunn et al., 2014). To date, there is a paucity of research identifying personal resources that could protect couples from the deleterious effects of sleep problems in either partner. To address this gap, the present study uses data from a longitudinal community lifespan sample of romantic partners to examine the influence of goal adjustment capacities (i.e., goal disengagement and goal reengagement; Wrosch et al., 2003) on the management of spousal sleep problems and relationship satisfaction, considering both partners perspectives. We reasoned that poor individual and spousal sleep are potent stressors that may require each partner to disengage from certain goals and redirect resources toward the effective management of their own or their partner's sleep problems. Consequently, we conducted exploratory analyses to examine if goal adjustment capacities could elicit coping responses in the context of sleep problems and, through this process, forecast relationship satisfaction.

### The role of goal adjustment capacities

Goal adjustment capacities refer to two independent personality dimensions associated with how people tend to respond to the occurrence of unattainable goals: goal disengagement and goal reengagement capacities (Wrosch et al., 2003; Wrosch and Scheier, 2020). Goal disengagement capacities reflect the tendency to withdraw commitment and effort from an unattainable goal, while goal reengagement capacities represent the tendency to identify, commit to, and pursue alternative goals when unattainable goals are encountered (Wrosch et al., 2003).

Goal adjustment capacities serve adaptive functions for subjective wellbeing if they prevent repeated failure and distress from life circumstances that render important goals unattainable (e.g., unemployment or a disease) and facilitate the engagement in new meaningful goals (e.g., learning new skills to obtain a job or live with a disease, Wrosch et al., 2003). In addition, goal adjustment may foster effective self-regulation when the pursuit of a goal is (temporarily) unfeasible due to emerging stressors that require people to abandon certain goals and redirect resources to the most relevant tasks (Wrosch et al., 2003). These capacities may thus determine the ways people cope with specific stressors (Lazarus and Folkman, 1984; for research on coping, see Carver et al., 1989) by triggering cognitive and behavioral strategies that promote subjective wellbeing (Wrosch et al., 2011; Wrosch and Sabiston, 2013).

In support of these assumptions, meta-analytic and longitudinal studies have shown that *goal disengagement capacities* facilitate subjective wellbeing (for meta-analysis, see Barlow et al., 2020). This line of research further suggests that goal disengagement capacities can enable a person to withdraw psychological and behavioral resources from unfeasible goals and reallocate resources to the management of pressing demands or the pursuit of other valuable activities (e.g., abandoning peripheral goals to care for a sick family member; Wrosch et al., 2003, 2011). *Goal reengagement capacities* have also been associated with subjective wellbeing (Barlow et al., 2020); however, their longitudinal effects can be adaptive or maladaptive. Beneficial consequences of goal reengagement have been observed if the pursuit of new goals is conducive to overcoming problems (e.g., exercise among cancer survivors; Wrosch and Sabiston, 2013). However, goal reengagement capacities can also be associated with a reduction of subjective wellbeing if the pursuit of new goals stretches a person too thin and prevents the person from addressing important stressors (Wrosch et al., 2011).

To date, research has only examined goal adjustment capacities at the level of the individual. However, a growing body of research highlights the importance of considering romantic partners in goal-related processes (e.g., Feeney, 2004; Gere and Impett, 2018; Holding et al., 2020; Katzenelenbogen et al., 2021; Zambrano et al., 2022). Further, existing dyadic frameworks underscore the interpersonal nature of stress and coping (Lindau et al., 2003; Berg and Upchurch, 2007; DeLongis et al., 2010; Helgeson et al., 2018). Therefore, we propose goal adjustment capacities could serve as an important resource in the face of individual and partner stressors. To avoid the negative consequences of these stressors, individuals and their partner's may need to abandon certain goals (e.g., work, leisure, or friendship goals) to free up resources needed for addressing the problems that underlie these stressors (e.g., sleep problems). Thus, goal disengagement capacities may serve an adaptive function by promoting effective coping responses in individuals and their partners, aimed at managing individual and partner stressors. Goal reengagement capacities, by contrast, may not necessarily exert the same beneficial effects. Although goal reengagement could facilitate the redirection of resources to overcoming these stressors, the pursuit of additional, new goals may deplete a person's resources, interfere with effective coping, and disturb relationship and personal wellbeing (Wrosch et al., 2011).

Preliminary support for the interpersonal benefits of goal adjustment can be found in research in a sample of parent caregivers of children with mental illness (Wrosch et al., 2011). This study found that while goal disengagement protected caregivers' subjective wellbeing, goal reengagement was associated with a reduction in subjective wellbeing. Interestingly, the effects of goal adjustment capacities were found to be partially explained by the care-specific coping strategies they elicited in the caregivers: the adaptive effects of goal disengagement were mediated by less self-blame and less substance use, while the maladaptive effects of goal reengagement were mediated by more venting and self-distraction. Further, a longitudinal study with community-dwelling older adults found that high levels of goal disengagement capacities protected older adults from the declines in social support satisfaction associated with declines in social support partners (Wrosch et al., 2013a,b). By contrast, low levels of goal reengagement capacities were associated with increases in social support satisfaction associated with increases in social support partners (Wrosch et al., 2013a,b). These results highlight that while goal disengagement capacities can facilitate coping with social stressors, the social benefits of goal reengagement depend on the resources available to the person. In this study, we extend this preliminary work by exploring the benefits of goal adjustment capacities in the context of sleep problems taking into account the perspectives of both members of the couple.

### A sample case: coping with sleep problems in romantic relationships

Population-based data from Canadian Community Health Survey suggests that ~50% of Canadians report sleep difficulties (Dai et al., 2020; Nunez et al., 2022). Sleep problems are often operationally defined by poor sleep efficiency (Carpenter and Andrykowski, 1998) and may result from exposure to stressors in different life domains (Âkerstedt, 2006). Sleep problems can also jeopardize goal pursuit (e.g., by interfering with performance; Centers for Disease Control Prevention, 2011) and predict adverse effects on psychological wellbeing (Dai et al., 2020; Nunez et al., 2022; Rodrigues et al., 2023). Further, a growing body of dyadic research highlights the interpersonal nature of sleep (e.g., Strawbridge et al., 2004; Troxel et al., 2007; Troxel, 2010; Gordon and Chen, 2014). Sleep in romantic couples is an inherently social process that is optimized when a person feels safe and secure with their partner, as sleep represents a behavioral state requiring the cessation of awareness and down-regulation of vigilance (Feeney and Kirkpatrick, 1996; Dahl and El-Sheikh, 2007). Further, if people share a bed with a partner and they are tossing and turning in your sleep, a partner's sleep may also be disrupted. Importantly, couples have been found to experience more conflict the day following a poor night's sleep (Gordon and Chen, 2014). Accordingly, sleep problems have been associated with reductions in relationship and martial satisfaction (Strawbridge et al., 2004; Troxel et al., 2007). Thus, sleep disturbances may not only jeopardize an persons' own relationship satisfaction but may affect their partner's relationship satisfaction as well.

To date, the majority of dyadic sleep research has focused on the adjustment of the person who experiences sleep problems, thereby disregarding the inherently social nature of this phenomenon in couples (Troxel et al., 2007). In addition, although population-based data suggests that ~50% of people report sleep difficulties (Dai et al., 2020; Nunez et al., 2022), much of the sleep literature has examined clinical populations (Strawbridge et al., 2004). Hence, relatively little is known about spousal sleep problems in community-dwelling populations (Strawbridge et al., 2004). Further, there is a lack of studies demonstrating the influence of personal resources that may alleviate the negative impact of individual and spousal sleep problems on relationship satisfaction. Such research, however, is crucial to better understand the personality dimensions that protect relationship satisfaction in the context of sub-clinical variation in sleep problems.

Considering that individual and partner sleep problems represent a stressor capable of interfering with individuals' own and their partners' goal pursuits and relationship satisfaction, we propose that goal adjustment capacities are an important resource that might influence this association. More specifically, goal disengagement capacities may allow people to abandon peripheral goals to free up resources needed for addressing the problems that underlie their own or their spouse's sleep problems. In contrast, goal reengagement capacities could facilitate the redirection of resources to overcoming peoples' own or their spouse's poor sleep due to problems in different life domains. As such the beneficial effects of goal adjustment capacities could be mediated by adaptive coping strategies. However, the pursuit of new goals may also overwhelm a person, thereby hindering successful coping and relationship functioning (Wrosch et al., 2011). In this scenario, goal reengagement might be associated with maladaptive coping and adverse outcomes.

### The present study

This longitudinal study examined the role of goal adjustment capacities and coping in the association between sleep efficiency and changes in relationship satisfaction in a community-dwelling lifespan sample of romantic couples. We hypothesized that poor spousal and own sleep would be longitudinally associated with a reduction of relationship satisfaction, but only if people report low (as compared to high) levels of goal disengagement capacities. Because of the potentially adaptive or maladaptive consequences of goal reengagement, we did not formulate a directed hypothesis for the role of goal reengagement capacities in psychological outcomes of sleep problems. Finally, we sought to explore whether emerging effects of both goal adjustment capacities on the associations between sleep problems and relationship satisfaction would be mediated by changes in relationship-specific coping responses among individuals and their spouses. Importantly, we did not have directed hypotheses about the role of specific coping strategies, therefore these analyses should be considered exploratory. In fact, the literature reviewed in the introduction suggested that goal adjustment capacities could elicit both adaptive and maladaptive coping strategies in the context of dyadic sleep problems.

## Methods

### Participants

The present study analyzed longitudinal data collected from a sample of 153 community-dwelling heterosexual couples (*n* = 306) from the greater Montreal area. These analyses used archival data from a larger study, therefore sample size was determined by data availability. Participants were recruited through newspaper advertisements. In order to participate in the study, couples had to be cohabitating and both partners had to be at least 18 years of age. Two waves of data were collected, ~1 year apart (*M* = 1.11, *SD* = 0.24). The final analytic sample included 113 couples (*n* = 226; *M*_*age*_ = 48.08; *SD*_*age*_ = 16.24). Data was excluded if both partners did not participate in the second wave (*n* = 70) or provide data on the outcome variable (*n* = 4) and goal adjustment measure (*n* = 6). Missing scores of single items were replaced during scale computation by the mean of available scores. All other missing scores of study variables were replaced with the sample mean (*n*_*sleep*_ = 5, *n*_*cope*_ = 2). The final sample did not differ from the baseline sample in terms of sleep efficiency, goal adjustment, and relationship satisfaction. However, excluded participants were younger (*t* = −4.36, *p* < 0.01), had a lower socioeconomic status (SES; *t* = −2.07, *p* = 0.04), and utilized higher levels of relationship-specific humor (*t* = 2.62, *p* = 0.01), substance abuse (*t* = 2.09, *p* = 0.04), and self-blame (*t* = 2.35, *p* = 0.02) coping strategies. Study attrition was not attributable to any other targeted study variable.

### Procedure

Participants completed a questionnaire at each study assessment in the laboratory. The questionnaire included measures of relationship satisfaction, sleep efficiency, goal adjustment capacities, relationship-specific coping strategies, and relevant control variables. After the completion of the questionnaire, all materials were collected. All materials have been made available on OSF. Participants were compensated $30 each for their participation in each of the study assessments. Informed consent was obtained from all participants prior to participation. The Concordia University Research Ethics Board approved all procedures and methods.

### Materials

Goal adjustment capacities were assessed at baseline using the 10-item Goal Adjustment Scale (GAS; Wrosch et al., 2003). These capacities refer to individual tendencies to react to the occurrence of unattainable goals across life domains and fall into two independent dimensions: goal disengagement capacities (four items) and goal reengagement capacities (six items). Participants were asked to indicate how they usually react if they can no longer pursue an important goal. Participants responded using 5-point Likert-type scales ranging from *strongly disagree* (1) to *strongly agree* (5). *Goal disengagement* capacities were indexed by computing a mean score of the four items that encompassed the tendency to reduce commitment and effort toward unattainable goals (e.g., “It's easy for me to reduce my effort toward the goal;” α = 0.75). *Goal reengagement* capacities were indexed by computing a mean score of the six items that encompassed the tendency to identify, commit to, and put effort toward new goals (e.g., “I start working on other new goals;” α = 0.87).

Sleep efficiency was assessed at baseline using the Brief Pittsburgh Sleep Quality Index (Buysse et al., 1989). Time in bed (A) was determined from participant's self-reports of the time they usually go to, and get out of bed. Sleep loss (B) was determined by computing a sum score of participants' reports of how many minutes they spent awake in bed before falling asleep, throughout the night, and before getting out of bed. Sleep efficiency was computed as a ratio of time spent asleep to time spent in bed [i.e., sleep efficiency = ((A–B)/A; Buysse et al., 1989].

Relationship satisfaction was assessed at both study assessments using the 7-item Relationship Assessment Scale (RAS, Hendrick, 1988). Participants were asked to respond to each question about their feelings toward their relationship over the past several weeks and months. Participants responded using 5-point Likert-type scales ranging from *not at all* (0) to *very much* (4). For each study assessment, relationship satisfaction was indexed as a mean score of the seven items (e.g., “In general, how satisfied are you with your relationship?” α_T1_ = 0.88; α_Ts_ = 0.90). Changes in relationship satisfaction were operationalized in a regression analysis as residualized change scores of relationship satisfaction at follow-up, controlling for relationship satisfaction at baseline. Relationship satisfaction was correlated between and within dyad members (T_1_: *r* = 0.53, *p* < 0.01; T_2_: *r* = 0.51, *p* < 0.01; ΔT_1_-T_2_: *r* = 0.31, *p* < 0.01) and mean levels of relationship satisfaction did not significantly differ between time points [*t*_(225)_ = −0.26, *p* = 0.80].

Relationship-specific coping strategies were assessed at both time points using the 28-item Brief Cope (Carver, 1997). Participants were asked to indicate how often they engaged in specific coping behaviors to manage stress and problems encountered in their intimate relationship. Participants responded using 4-point Likert-type scales ranging from *I haven't been doing this at all* (0) to *I've been doing this a lot* (3). For each study assessment, each of the 14 types of coping strategies were indexed as a mean score of the two associated items (see OSF for complete measure). Across both assessments, the two items representing each coping strategy were consistently positively correlated (all *r*s = 0.24 to 0.91; all *p*s < 0.01). Changes in each relationship-specific coping strategy were operationalized as residualized change scores of the coping strategy at follow-up, controlling for the coping strategy at baseline.

Covariates were assessed at baseline to avoid spurious effects associated with variables that could affect sleep. Self-reports of participants' age, sex, education level (0 = no education, 1 = high school, 2 = college diploma, 3 = bachelor's degree, 4 = master's or doctoral degree), and annual family income (0 = < $17,000, 1 = up to $34,000, 2 = up to $51,000, 3 = up to $68,000, 4 = up to $85,000, 5 = more than $85,000) were obtained. Self-reported education level and annual family income were assessed to index SES (*r* = 0.23, *p* < 0.01). Their standardized scores were averaged to obtain a reliable measure of SES.

### Data analyses

The data and syntax for this study is available on OSF. Prior to data analyses, all variables were screened for outliers and distributional characteristics. Outliers were replaced by the most extreme value within the normal distribution. Subsequently, all main variables demonstrated a normal distribution (*skewness* < |1.38|, *kurtosis* < |1.68|). Preliminary analyses were conducted to describe the sample and examine correlations between variables. The study's main hypotheses were subsequently tested using hierarchical linear modeling (MIXED procedure in SPSS) with individuals nested within couples. More specifically, we estimated an actor-partner interdependence model with moderation (Garcia et al., 2015). Sex was not included into the model since the model were found to be indistinguishable by sex, $X_{\left(9\right)}^{2}$ = 9.84, *p* = 0.36, implying that reported effects were not found to be significantly different between men and women. Accordingly, the reported effects were pooled across all participants (men and women) as both, actors and partners. Further, partner goal adjustment capacities did not add significantly to the model, $X_{\left(6\right)}^{2}$ = 2.28, *p* = 0.89, and therefore were not included in the models for reasons of parsimony. All analyses controlled for actor age and SES.

First, all main predictor variables were added to the Level-1 model. Predictor variables were grand-mean centered prior to the analysis. This model examined the main effects of actor sleep efficiency, partner sleep efficiency, actor goal disengagement, and actor goal reengagement in predicting changes in the actor's relationship satisfaction. The effect coefficients can be interpreted as the residualized change in relationship satisfaction given a one-unit increase in the predictor. Second, the four interaction terms between sleep efficiency (actor or partner) and actor goal adjustment (goal disengagement or goal reengagement) were added to the Level-1 model. This step examined the moderating effect of actor goal adjustment capacities on the relation between actor and partner sleep efficiency and changes in actor's relationship satisfaction. The Level-2 models did not include additional variables. Due to the limited number of degrees of freedom (i.e., only two dyad members), Level-2 models estimated only fixed effects of the Level-1 predictors, except the intercept (i.e., only the intercept was allowed to vary between dyad members). Significant interaction effects were followed up by estimating the simple slopes of the association between actor or partner sleep efficiency and changes in the actor's outcome, separately for actors who reported low or high levels of goal adjustment capacities, using −1 SD and +1 SD of the predictor variables as reference points (Preacher et al., 2006).

Further, exploratory mediation analyses were conducted by estimating the confidence intervals of the indirect effects using parametric bootstrapping via the Monte Carlo Method (Selig and Preacher, 2008) to determine if the significant interaction effects were mediated by changes in specific actor or partner coping strategies. Since the authors of the COPE did not suggest aggregating different coping strategies, but recognize empirical overlap among specific coping strategies, we chose a two-step procedure (Scheier et al., 1986; Carver et al., 1989). First, we examined the correlations between changes in actor and partner coping strategies with the study outcome to identify potential mediators. Second, we conducted separate dyadic mediation analyses (i.e., one for each coping strategy identified) to pinpoint actor or partner coping strategies that mediated the observed interaction effects. Each separate mediation analysis included values for changes in both actor and partner coping strategy use.

## Results

### Preliminary analyses

Participants had a wide age range (21–82 years) and were on average 48 years old. The sample was collected to include relatively equal representation across the lifespan: 30.1% aged 21–35, 30.5% aged 36–55, and 39.4% aged 56–82. Most of the sample obtained a university degree. The average annual income was below $51,001. The average sleep efficiency was ~86%, consistent with sleep efficiency observed in lifespan samples (Ohayon et al., 2004). Refer to Tables 1, 2 for descriptive and correlation tables for the main study variables. Descriptives and correlation for the relationship specific coping strategies (Supplementary Tables 1–3), and all supplemental analyses are in the online Supplementary material on OSF.

**Table 1:** Means, standard deviations, and frequencies of main study variables (*n* = 226).

**Constructs**	**Mean (SD) or percentage**	**Range**
**Relationship satisfaction**		
T1	3.20 (0.64)	1.43–4
T2	3.19 (0.70)	1.14–4
Sleep efficiency (T1)	0.86 (0.12)	0.50–1
Goal disengagement (T1)	2.67 (0.84)	1–5
Goal reengagement (T1)	3.70 (0.75)	1.67–5
Age (T1)	48.08 (16.24)	21–82
**Education (%) (T1)**		
Other	0.9	
High school	22.6	
College	16.6	
Bachelor	33.2	
Master/Ph.D.	26.7	
**Annual income (%) (T1)**		
Less than $17,000	19.3	
$17,001–$34,000	19.7	
$34,001–$51,000	13.5	
$51,001–$68,000	15.2	
$68,001–$85,000	14.8	
>$85,000	17.5	

**Table 2:** Zero-order correlations between main study variables (*n* = 226).

	**1**	**2**	**3**	**4**	**5**	**6**	**7**
1. Actor relationship satisfaction (T1)							
2. Actor relationship satisfaction (T2)	0.77^**^						
3. Actor sleep efficiency	0.17^*^	0.15^*^					
4. Partner sleep efficiency	0.12	0.13^*^	0.16^*^				
5. Actor goal disengagement	0.03	0.05	−0.00	−0.03			
6. Actor goal reengagement	0.17^*^	0.12	−0.04	0.03	0.41^**^		
7. Age	−0.05	−0.06	−0.06	−0.08	0.09	−0.11	
8. Socioeconomic status	0.16^*^	0.13^*^	0.05	−0.02	0.08	0.18^**^	0.15^*^

^*^p < 0.05; ^**^p < 0.01.

### Goal adjustment capacities, sleep efficiency, and relationship satisfaction

Table 3 summarizes the results of a hierarchical linear model predicting changes in actor relationship satisfaction by actor and partner sleep efficiency, actor goal adjustment capacities, and the interactions between these variables. The specified models were found to fit the data better than an empty model [$X_{\left(10\right)}^{2}$ = 26.17, *p* < 0.01], explaining 7.17% of the variability in changes in relationship satisfaction (calculated as Pseudo R-squared values; see Kenny et al., 2006). The main effect model revealed no significant main effects on changes in relationship satisfaction. In the second step, two significant interactions emerged (Table 3).

**Table 3:** Actor-partner interdependence model with moderation predicting changes in relationship satisfaction (*n* = 226).

	Δ **actor relationship satisfaction**			
	***B***	***SE***	***t***	***p***
**Covariates**				
Age	−0.00	0.00	−0.96	0.34
Socioeconomic status	−0.01	0.04	−0.14	0.89
**Main effects**				
Actor sleep efficiency	0.04	0.23	0.19	0.85
Partner sleep efficiency	0.04	0.23	0.16	0.87
Actor goal disengagement (GD)	0.02	0.03	0.64	0.53
Actor goal reengagement (GR)	−0.00	0.04	−0.05	0.96
**Interactions**				
Actor sleep efficiency X GD	−0.11	0.28	−0.40	0.69
Actor sleep efficiency X GR	1.19	0.31	3.90	< 0.01
Partner sleep efficiency X GD	−0.65	0.27	−2.42	0.02
Partner sleep efficiency X GR	0.49	0.30	1.65	0.10

Degrees of freedom are empirically adjusted for similarity in scores between dyad members (range: 112.00–210.40). This table reports the parameter estimates for the covariate and main effects from the first model (i.e., without the interactions), and the interaction effects from the second model (i.e., controlling for all covariate and main effects).

**Figure 1:**
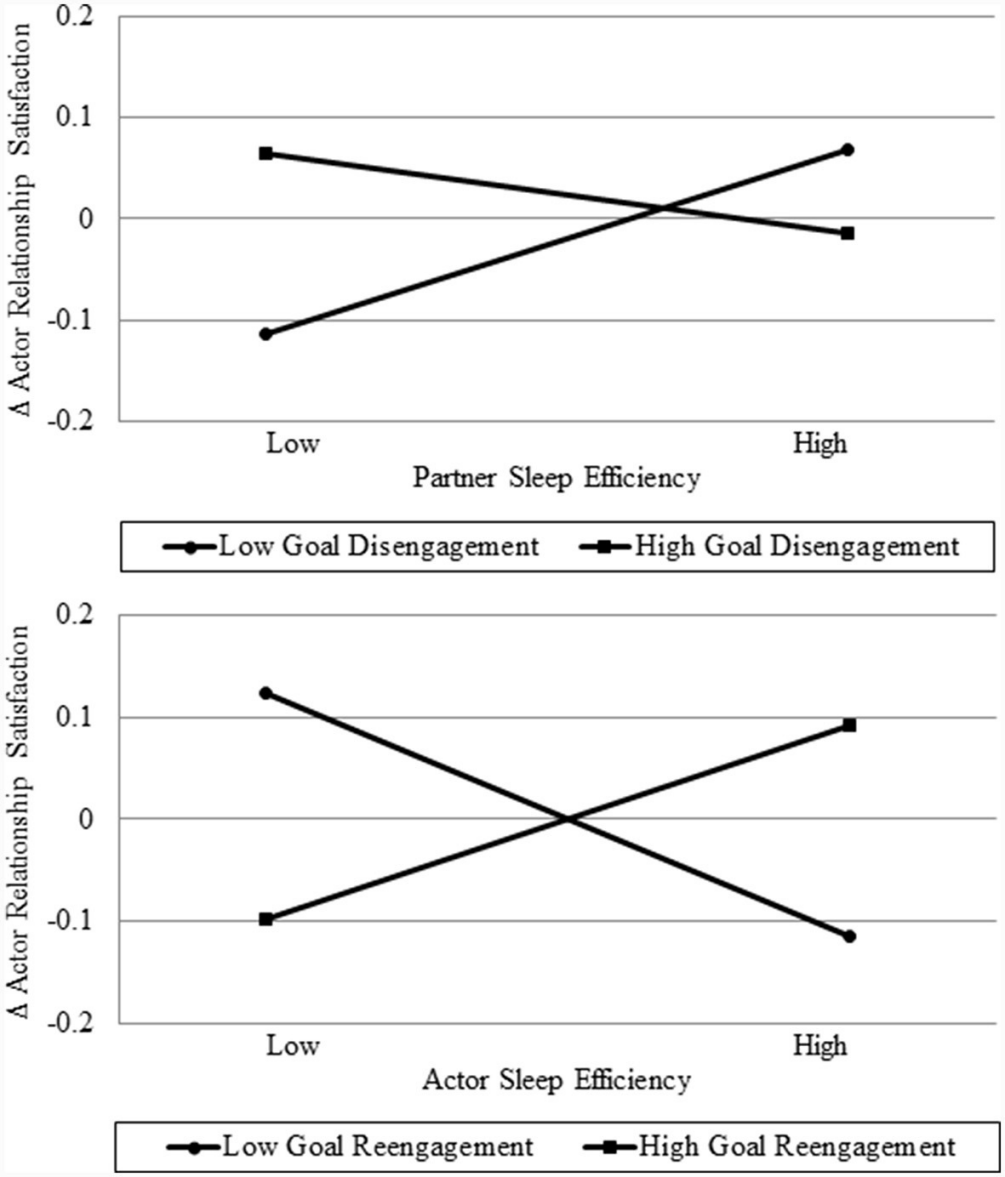
The relation between partner **(top panel)** and actor **(bottom panel)** sleep efficiency (+/−1 SD) and actor changes in relationship satisfaction for actors with low (−1 SD), and high (+1 SD) levels of goal disengagement **(top panel)** and goal reengagement **(bottom panel)** capacities.

First, the interaction between actor goal disengagement capacities and partner sleep efficiency was found to predict changes in relationship satisfaction [95% CI (−1.17, −0.12)]. This interaction effect is plotted in the top panel of Figure 1. Simple slope analyses indicated that the adverse effects of poor partner sleep efficiency on relationship satisfaction were stronger among participants with relatively low (*coefficient* = 0.76, *SE* = 0.34, *p* = 0.02), as compared to high levels of goal disengagement capacities (*coefficient* = −0.33, *SE* = 0.30, *p* = 0.28).

Second, the interaction between actor goal reengagement capacities and actor sleep efficiency was related to changes in relationship satisfaction [95% CI (0.59, 1.79)]. This interaction is plotted in the bottom panel of Figure 1. Simple slope analyses confirmed that the adverse effects of poor actor sleep efficiency on relationship satisfaction were stronger among participants with relatively high (*coefficient* = 0.79, *SE* = 0.34, *p* = 0.01), as compared to low levels of goal reengagement capacities (*coefficient* = −0.99, *SE* = 0.34, *p* < 0.01).

### The mediating role of coping

The preliminary correlational analyses of the associations between coping and changes in relationship satisfaction (Supplementary Table 4) point to changes in active coping (*r*_*actor*_ = 0.22, *r*_*partner*_ = 0.11), self-blame (*r*_*actor*_ = −0.17, *r*_*partner*_ = −0.25), behavioral disengagement (*r*_*actor*_ = −0.26, *r*_*partner*_ = −0.24), instrumental support (*r*_*actor*_ = 0.08, *r*_*partner*_ = 0.17), and denial (*r*_*actor*_ = −0.19, *r*_*partner*_ = −0.22) as potential mediators of the interaction effects on relationship satisfaction.

The main results of the exploratory mediation analyses are summarized in Supplementary Table 5. Outputs for all mediations analyses are available on OSF. Our mediation models examining the role of coping strategies in the interaction effect between actor goal disengagement and partner sleep efficiency demonstrated that changes in actor active coping [95% CI (−0.32, −0.02)] and partner self-blame coping [95% CI (−0.28, −0.01)] exerted a significant indirect effect on the relation between the interaction effect and changes in relationship satisfaction. Further, these indirect effects held when controlling for each other in a single model [actor active coping: 95% CI (−0.32, −0.02); partner self-blame: 95% CI (−0.28, −0.01)]. Results further showed that the interaction between partner sleep efficiency and goal disengagement was associated with changes in actor active coping (*B* = −1.10, *SE* = 0.39, *p* < 0.01), and partner self-blame (*B* = 0.93, *SE* = 0.41, *p* = 0.02). Simple slopes analyses revealed that poorer partner sleep efficiency was more strongly associated with adaptive coping responses among participants with relatively high levels of goal disengagement (actor active coping: *B* = −0.59, *SE* = 0.44, *p* = 0.18; partner self-blame: *B* = 0.43, *SE* = 0.45, *p* = 0.34), as compared to low levels of goal disengagement capacities (actor active coping: *B* = 1.25, *SE* = 0.49, *p* < 0.01; partner self-blame: *B* = −1.13, *SE* = 0.50, *p* = 0.03). In addition, increases in actor active coping were associated with enhanced relationship satisfaction (*B* = 0.13, *SE* = 0.05, *p* < 0.01), while increases in partner self-blame were associated with decreased relationship satisfaction (*B* = −0.13, *SE* = 0.04, *p* < 0.01).

The mediation analyses examining the role of coping strategies in the interaction effect between actor goal reengagement and actor sleep efficiency demonstrated that changes in actor behavioral disengagement coping [95% CI (0.05, 0.41)] exerted significant indirect effects on the relation between the interaction effect and changes in relationship satisfaction. The results further showed that the interaction between actor sleep efficiency and goal reengagement was associated with changes in actor behavioral disengagement (*B* = −1.15, *SE* = 0.39, *p* < 0.01). Simple slopes analyses revealed that poorer actor sleep efficiency was more strongly associated with maladaptive coping responses among participants with relatively high levels of goal reengagement (*B* = −0.70, *SE* = 0.39, *p* = 0.07), as compared to low levels of goal reengagement capacities (*B* = 1.03, *SE* = 0.43, *p* = 0.02). Finally, increases in actor behavioral disengagement were associated with decreases in relationship satisfaction (*B* = −0.18, *SE* = 0.05, *p* < 0.01).

## Discussion

The present study extends past individual-focused research by embracing the social context of goal adjustment using the sample case of sleep problems in cohabitating romantic partners. Findings document in a sample of couples across the adult lifespan that higher, as compared to lower, levels of goal disengagement capacities, can prevent reduced relationship satisfaction among people whose spouse experiences inefficient sleep. Conversely, higher, as compared to lower, levels of goal reengagement capacities were shown to enhance the adverse effect of poor individual sleep efficiency on relationship satisfaction. Further, the observed effects of goal adjustment capacities were mediated by engagement in specific coping strategies targeted at addressing with relationship problems. These findings support dyadic frameworks, pointing to the interpersonal nature of stress, coping, and wellbeing (Lindau et al., 2003; Berg and Upchurch, 2007) by demonstrating individual and spousal effects of stress experiences and self-regulation processes in predicting relationship satisfaction. More specifically, they suggest that goal disengagement capacities and associated coping responses (i.e., more actor active coping and less partner self-blame) represent important psychological factors for preserving relationship satisfaction in the context of spousal sleep problems. Conversely, goal reengagement capacities may trigger coping strategies that enhance adverse effects of poor sleep on relationship satisfaction (i.e., more actor behavioral disengagement).

We suggest that goal disengagement capacities buffered the effects of spousal sleep problems on reduced relationship satisfaction because these capacities enable a person to redirect resources to the management of pressing demands, and thus contribute to the avoidance of continuing problems (Wrosch et al., 2003, 2013a,b). In this way, people with relatively low levels of goal disengagement capacities may fail in abandoning peripheral goals (e.g., work, leisure, or friendship goals), and experience difficulties with reallocating resources (e.g., time and energy) to the management of a stressor, such as spousal sleep problems. Consequently, sources underlying sleep problems may not be addressed, resulting in a perpetuation of stressful experiences, and consequently compromising relationship satisfaction. Conversely, people with relatively high levels of goal disengagement capacities may be better able to reallocate the resources necessary for overcoming the spousal stressor and may be protected from experiencing diminished relationship satisfaction.

The study's findings further showed that an increase in specific coping tactics aimed at directing resources toward the relationship (i.e., through actors' active coping) and self-protective coping responses among their spouses (i.e., by spousal avoidance of self-blame) mediated the observed buffering effects of goal disengagement capacities on relationship satisfaction. Such a process may occur if high levels of goal disengagement capacities increase a person's own active coping tactics aimed at overcoming spousal sleep problems, which is likely to resolve relationship stressors and protect relationship satisfaction. By contrast, people with low levels of goal disengagement capacities may not enhance their active coping efforts in the context of spousal sleep problems, and therefore experience reduced relationship satisfaction. Further, spouses with poor sleep increased their levels of self-blame for relationship problems and experienced a reduction in relationship satisfaction, but only if their partners reported low (but not high) levels of goal disengagement capacities. This pattern of effects may imply that people with poor goal disengagement capacities could struggle with maintaining the pursuit of multiple goals in addition to dealing with a relationship stressor, which may increase their partner's feelings of responsibility for occurring problems and compromise their relationship satisfaction.

Different from the effects for goal disengagement capacities, high levels of goal reengagement capacities were found to be associated with reduced relationship satisfaction in response to poor own sleep. These results may be explained by research, documenting adaptive and maladaptive effects of goal reengagement capacities (Wrosch et al., 2011, 2013a,b). Supposedly, the adaptive value of goal reengagement capacities depends on the extent to which the adoption of new goals is conducive or harmful to overcoming a stressful situation. In the context of poor sleep, new goals could stretch a person too thin, create additional problems, and result in compromised relationship satisfaction.

The results document that the adverse effects of goal reengagement capacities were mediated by changes in individuals' engagement in relationship-specific behavioral disengagement. Relatively high levels of goal reengagement capacities were associated with stronger increases in the use of relationship-specific behavioral disengagement in response to poor own sleep. In addition, increased levels of actor behavioral disengagement were related to reduced relationship satisfaction. This indirect association lends further support to the possibility that goal reengagement capacities may have resulted in the adoption of new goals outside the relationship that are not conducive to the effective self-regulation of sleeping problems among couples. For example, the pursuit of new goals outside the relationship may require people to withdraw behavioral efforts from the relationship, thereby jeopardizing relationship satisfaction.

The presented findings have important implications for theory and research. First, they contribute to the literature on goal adjustment by expanding past research on the psychological consequences of goal adjustment to the interpersonal context (Wrosch et al., 2011, 2013a,b). Our study demonstrates that goal disengagement, but not goal reengagement, capacities represent an important personal resource capable of eliciting adaptive behavioral and psychological coping responses when people are confronted with spousal stressors. More specifically, the reported results suggest that goal disengagement capacities may contribute to improved relationship satisfaction by freeing up resources that can be used to address stressors experienced by a person's spouse. Conversely, goal reengagement capacities may compromise relationship satisfaction by stretching a person's resources too thin, in turn compromising the relationship satisfaction by pulling resources away from addressing relationship-specific stressors. These findings support this conclusion by documenting that the psychological consequences of individuals' goal adjustment capacities were related to changes in relationship-specific coping patterns. Here, the study's findings showed that goal disengagement capacities elicited their effects by fostering adaptive coping responses among individuals and their spouses, while goal reengagement's adverse effects were related to the use of maladaptive coping responses. Thus, goal disengagement and goal reengagement capacities play important, but opposing, roles in romantic relationships, as they may facilitate or hinder the resolution of spousal stressors.

Second, the present study expands the psychological literature on dyadic sleep in different ways. Most sleep research has focused on the adjustment of the individual who experiences sleep problems (Troxel et al., 2007). The present study extends this line of work by drawing on a theoretical rationale that examines sleep problems as a phenomenon impacting the entire dyad, and demonstrating the social dynamics of stress, coping, and relationship satisfaction in the context of sleep. In addition, dyadic sleep research has largely focused on clinical samples (Troxel et al., 2007) and studies on community dwelling populations are scarce and often cross-sectional in nature (Strawbridge et al., 2004). Our research contributes to this literature by examining in longer-term longitudinal analyses the temporal order of associations between sleep problems and relationship satisfaction. The conclusions derived from this approach suggest that spousal sleep problems can be associated with declines of an individual's relationship satisfaction, unless they possess the personal resources needed to effectively cope with this spousal stressor. To this end, the identification of goal adjustment capacities as influential personal resources may contribute to broadening the search for other personality dimensions that could exert similar effects.

Finally, the study's results have implications for psychological interventions. Given the reciprocal associations between relationship satisfaction and sleep problems (Hasler and Troxel, 2010), the present study highlights the possibility that couples could enter a downward spiral, in which effects of poor sleep on relationship satisfaction result in a continued deterioration of quality of life. Based on the presented findings, it may be possible for clinicians to prevent such an adverse cascade by identifying couples with poor sleep and low levels of goal disengagement capacities, or high levels of goal reengagement capacities, and working with them on a withdrawing commitment and efforts from more peripheral, specific goals to redirect resources to the management of spousal stressors. Such interventions may foster effective coping with spousal stressors and contribute to wellbeing in romantic relationships.

## Limitations and future directions

The present study has limitations that should be addressed in future research. First, the data stem from a relatively small longitudinal sample of cohabitating couples examined over a relatively short period of time (i.e., 1 year), limiting the generalizability of the findings. Further, it is possible that recruiting participants through newspaper advertisement might have resulted in a selection bias toward a more educated sample. However, our data are consistent with past research demonstrating concordance of sleeping patterns and relationship satisfaction among romantic partners (Gunn et al., 2014). Nonetheless, replication in larger, long-term, and population-based studies is warranted.

Second, the study's methodology does not permit us to draw causal conclusions. For example, it may be that some people foresee declines in relationship satisfaction in their future, which could impact present sleep patterns in romantic relationships. Future studies could shed light on causal relations among other aspects of the proposed theoretical model by fostering disengagement from certain specific goals and associated coping patterns in intervention studies with couples who experience sleep problems.

Third, the present study did not provide information on participants' specific goals and thus could not identify the concrete goals that people with high goal adjustment capacities may abandon in the context of spousal sleep problems. Future research should assess such information to illuminate the goal-specific processes that contribute to the interpersonal benefits of goal disengagement capacities, and prevent people with high goal reengagement capacities from experiencing the same adaptive outcomes.

Finally, given that changes in coping and relationship satisfaction overlapped in our study, it is difficult to tease apart the directional relations between these constructs. Although our theoretical rationale is based on personality theories that postulate coping strategies to influence wellbeing (e.g., Lazarus and Folkman, 1984; Carver et al., 1989), these associations could be reciprocal. In a similar vein, it seems reasonable to assume that decreased relationship satisfaction could spill over and affect a general reduction of emotional wellbeing and physical health. This is in line work demonstrating the deleterious effects of sleep problems on subjective wellbeing and physical health (Revenson et al., 2016; Troxel et al., 2017; Uchino et al., 2019). Future research should thus utilize long-term longitudinal designs to disentangle the directional processes between coping responses and relationship satisfaction among dyads who experience poor sleep, and extend this model to indicators of subjective wellbeing and physical health.

## Conclusions

This lifespan study of romantic couples demonstrates that goal disengagement capacities can protect individuals whose spouses experience poor sleep from reductions in relationship satisfaction. Goal reengagement capacities, by contrast, were less adaptive if individuals experienced poor sleep. These longitudinal associations were mediated by changes in the use of adaptive vs. maladaptive coping strategies among individuals and their spouses. The study's findings highlight the importance of individual differences in self-regulation tendencies and associated coping tactics for the management of individual and spousal stressors in romantic relationships.

## Data availability statement

The original contributions presented in the study are included in the article/Supplementary material, further inquiries can be directed to the corresponding author.

## Ethics statement

This study involving humans was approved by Concordia University Research Ethics Board. The study was conducted in accordance with the local legislation and institutional requirements. The participants provided their written informed consent to participate in this study.

## Author contributions

MB: Conceptualization, Data curation, Formal analysis, Methodology, Visualization, Writing – original draft, Writing – review & editing. CW: Conceptualization, Funding acquisition, Supervision, Writing – review & editing. CH: Conceptualization, Funding acquisition, Writing – review & editing.
