# Supplementary material for: The interpersonal benefits of goal adjustment capacities: the sample case of coping with poor sleep in couples
Source: Front Psychol. 2024 Mar 19;15:1287470. doi: 10.3389/fpsyg.2024.1287470 (PMC10985202; doi:10.3389/fpsyg.2024.1287470)
Supplement: Supplementary file 1 [file Table_1.docx]

Table OSM 1.

*Means and Standard Deviations of Relationship-Specific Coping Scales*

|  | T1 | |  | T2 | |
| --- | --- | --- | --- | --- | --- |
| Constructs | Mean | SD |  | Mean | SD |
| Active  Self-Blame  Behavioral Disengagement  Positive Reappraisal  Humor  Emotional Support  Instrumental Support  Self-Distraction  Denial  Venting  Planning  Acceptance  Religion  Substance Abuse | 1.98  1.05  0.47  1.71  0.93  1.03  1.04  1.44  0.42  1.51  1.94  1.59  1.11  0.24 | 0.63  0.77  0.64  0.72  0.79  0.87  0.81  0.77  0.62  0.76  0.75  0.78  1.10  0.60 |  | 1.98  1.03  0.38  1.76  1.00  0.94  0.96  1.35  0.33  1.36  1.79  1.52  1.06  0.21 | 0.67  0.70  0.57  0.74  0.82  0.84  0.82  0.77  0.58  0.81  0.80  0.81  1.07  0.54 |

Table OSM 2.

*Zero-Order Correlations Between Changes in Actor Relationship-Specific Coping Scales and Main Study Variables*

| Δ Coping Strategy |  | 1 | 2 | 3 | 4 | 5 | 6 | 7 | 8 |
| --- | --- | --- | --- | --- | --- | --- | --- | --- | --- |
| Active  Self-Blame  Behavioral Disengagement  Positive Reappraisal  Humor  Emotional Support  Instrumental Support  Self-Distraction  Denial  Venting  Planning  Acceptance  Religion  Substance Abuse |  | .00  -.13  -.12  -.01  .11  .03  -.01  -.09  -.24  .02  .15  .04  .00  -.02 | .13  -.23  -.26  .06  .16  .08  .06  -.13  -.30  -.01  .16  .03  .05  .00 | -.06  -.04  .01  .03  .05  .03  -.02  .04  -.13  -.03  -.06  .12  -.15  -.04 | .04  -.10  -.07  -.11  .06  -.03  -.02  .04  .03  .13  -.05  -.08  .03  -.10 | -.11  -.14  -.09  -.05  -.08  -.07  -.09  -.15  -.02  -.05  -.11  -.11  -.12  -.07 | .15  .06  .01  .01  -.05  .00  -.01  -.03  .08  .03  .04  .10  .04  .07 | -.08  -.11  -.13  .00  -.07  -.15  -.11  -.10  .05  -.04  .00  -.01  -.11  .03 | -.04  -.15  -.16  -.05  -.09  -.12  -.11  -.06  -.04  -.03  .01  .04  -.13  .06 |

*Note.* 1 = Actor relationship satisfaction (T1); 2 = Actor relationship satisfaction (T2); 3 = Actor sleep efficiency; 4 = Partner sleep efficiency; 5 = Actor goal disengagement; 6 = Actor goal reengagement; 7 = Age; 8 = Socioeconomic status.

Table OSM 3.

*Zero-Order Correlations Between Changes in Partner Relationship-Specific Coping Scales and Main Study Variables*

| Δ Coping Strategy |  | 1 | 2 | 3 | 4 | 5 | 6 | 7 | 8 |
| --- | --- | --- | --- | --- | --- | --- | --- | --- | --- |
| Active  Self-Blame  Behavioral Disengagement  Positive Reappraisal  Humor  Emotional Support  Instrumental Support  Self-Distraction  Denial  Venting  Planning  Acceptance  Religion  Substance Abuse |  | .06  -.13  -.10  -.03  .01  .08  -.01  -.03  -.13  .01  .11  .12  .00  .00 | .14  -.27  -.24  .07  .03  .12  .10  -.03  -.24  .04  .17  .06  -.01  .05 | .04  -.10  -.07  -.11  .06  -.03  -.02  .04  .03  .13  -.05  -.08  .03  -.10 | -.06  -.04  .01  .03  .05  .03  -.02  .04  -.13  -.03  -.06  .12  -.15  -.04 | .05  -.01  -.05  .05  -.02  -.02  -.05  -.04  .03  -.06  .01  -.02  .01  .00 | .03  .08  .01  .07  .06  .00  -.04  -.03  .00  .03  .06  -.02  .05  -.08 | -.07  -.12  -.10  -.02  -.04  -.10  -.10  -.09  .09  .01  .00  .00  -.11  .04 | .03  -.07  -.17  .03  -.06  -.04  -.08  -.04  .05  -.03  .08  -.03  -.11  -.01 |

*Note.* 1 = Actor relationship satisfaction (T1); 2 = Actor relationship satisfaction (T2); 3 = Actor sleep efficiency; 4 = Partner sleep efficiency; 5 = Actor goal disengagement; 6 = Actor goal reengagement; 7 = Age; 8 = Socioeconomic status.

Table OSM 4.

*Zero-Order Correlations Between Changes in Coping Strategies and Relationship Satisfaction*

|  |  | Δ Relationship Satisfaction | |
| --- | --- | --- | --- |
| Δ Coping Strategy |  | Actor | Partner |
| Active |  | .219** | .110 |
| Self-Blame |  | -.167* | -.245** |
| Behavioral Disengagement |  | -.262** | -.240** |
| Positive Reappraisal |  | .118 | .118 |
| Humor |  | .127 | .013 |
| Emotional Support |  | .079 | .080 |
| Instrumental Support |  | .083 | .165* |
| Self-Distraction |  | -.099 | -.014 |
| Denial |  | -.186** | -.221** |
| Venting |  | -.044 | .041 |
| Planning |  | .081 | .108 |
| Acceptance |  | .005 | -.057 |
| Religion |  | .083 | .012 |
| Substance Abuse |  | .017 | .092 |

* *p* < .05; ** *p* < .01

Table OSM 5.

*Dyadic Mediation Results: 95% Confidence Intervals of the Indirect Effects*

|  |  | Δ Actor Relationship Satisfaction | | |
| --- | --- | --- | --- | --- |
| Δ Coping Strategy | | Partner Sleep x  Goal Disengagement |  | Actor Sleep x  Goal Reengagement |
| Active | Actor  Partner | **-0.319, -0.023**  -0.156, 0.021 |  | -0.009, 0.269  -0.069, 0.100 |
| Self-Blame | Actor  Partner | -0.135, 0.040  **-0.278, -0.010** |  | -0.135, 0.062  -0.014, 0.268 |
| Behavioral Disengagement | Actor  Partner | -0.156, 0.104  -0.163, 0.127 |  | **0.048, 0.412**  -0.229, 0.102 |
| Instrumental Support | Actor  Partner | -0.081, 0.035  -0.110, 0.047 |  | -0.102, 0.039  -0.019, 0.186 |
| Denial | Actor  Partner | -0.016, 0.201  -0.114, 0.135 |  | -0.028, 0.208  -0.128, 0.156 |

*Note*. A separate model was estimated for each coping strategy with both actor and partner measures included. Significant effects are bolded.
